# Implant Diameter, Length, and the Insertion Torque/Depth Integral: A Study Using Polyurethane Foam Blocks

**DOI:** 10.3390/dj8020056

**Published:** 2020-06-04

**Authors:** Paolo Arosio, Federico Arosio, Danilo Alessio Di Stefano

**Affiliations:** 1 Private Practitioner, Vimercate, 20871 Monza-Brianza, Italy; p.arosio@libero.it; 2 Dental School, University of Milan, 20122 Milan, Italy; fedix_8@hotmail.it; 3 Adjunct Professor, Dental School, Vita-Salute University IRCCS San Raffaele, 20132 Milan, Italy; 4 Private Practitioner, 20148 Milan, Italy

**Keywords:** primary stability, torque-depth curve integral, insertion torque, undersizing, fixture length, fixture diameter

## Abstract

The amount of energy necessary to place an implant in its seat, described as the integral of the torque-depth curve at insertion (I), has been validated as a reliable measure of primary stability. This study aimed to investigate whether (I) may detect the variations in primary stability caused by changes in the implant length or diameter better than the insertion torque (IT). Cylindric implants featuring a double-etched, sandblasted surface with different diameters or lengths were placed into monolithic polyurethane foam blocks with different densities that mimicked human bone. (I)-, (I)*-, IT-, IT*-diameter and -length plots ((I)* and IT* were the derived values corrected for undersizing) were drawn and the relation between (I), (I)*, IT, and IT* and the fixture diameter or length was investigated with correlation analysis. (I)* and IT* correlated better than (I) and IT with the fixture diameter; (I), (I)*, IT, and IT* correlated equally well with the fixture length. In all cases, the slopes of the lines best fitting the experimental data were greater for (I) or (I)* than IT or IT*, respectively. (I) or (I)* were better detectors than IT or IT* of the changes in primary stability that can be achieved by increasing the fixture diameter or length.

## 1. Introduction

Primary implant stability is one of the determinants of implant success, as micromovements exceeding the 50–150 µm threshold may hinder osseointegration and lead to implant failure [1,2,3,4]. Further, certain primary stability thresholds are necessary to apply early or immediate after loading in order to rehabilitate the patient earlier. There are many factors affecting primary stability [4,5]. Some are patient-related, such as the bone density at the insertion site or the thickness of the cortical bone layer; others include the micro- and macro-morphology of the implant being placed. The extent of under preparation in low-density sites or of tapping in high-density sites are other operative choices. The implant diameter and length are among the first parameters the surgeon chooses when planning the whole rehabilitation process [6,7]. Fully understanding how these parameters may affect primary stability is therefore of paramount importance. When the effect of these two parameters on primary stability were investigated in a clinical setting, the results were inconclusive. When Degidi et al. [8] retrospectively assessed 4135 implants in 1045 consecutive patients, they did not find any significant correlation between the implant diameter and the insertion torque (IT), nor between the implant length and IT; they observed the implant stability quotient (ISQ), measured with resonance frequency analysis (RFA), to correlate with the implant length, but they observed no correlation between the ISQ and the implant diameter. Mesa et al. [9] retrospectively assessed the primary stability of 1084 implants placed in 316 patients. The Periotest Value (PTV) was used for measurement and they found it was significantly associated with implant length, but not with implant diameter. Ostman et al. [10] retrospectively assessed 905 implants in 267 patients and found that their ISQ correlated significantly with both their diameter and length. Aragoneses et al. [11] prospectively placed 559 implants in 159 patients and found no correlation between the ISQ and their diameter. Schiffler et al. [12] retrospectively assessed 200 implants in 143 patients and observed that their ISQ did not correlate with the implant diameter or its length. Similar varied observations were reported by other authors [13,14,15,16,17,18,19,20,21,22,23,24]. Some of this discordance may arise from the effect of confounding factors; indeed, in the studies mentioned above, different implant brands and models were used, the insertion protocol was either that provided by the manufacturer or a different protocol was used, and implants were placed in different zones of the two arches. Yet, some authors have also highlighted how different primary stability measuring parameters (e.g., IT, ISQ, and PTV) actually measure different physical quantities. For example, Degidi et al. [8] found no correlation between IT and ISQ values at insertion and explained this finding by remarking how IT is correlated to rotational friction and ISQ to lateral displacement [25]. It follows that changes in the implant diameter or length may affect primary stability differently, and results of studies using different parameters may therefore be inconsistent. Indeed, such inconsistency in results concerning the effect of the implant diameter and length on different primary stability parameters has already been observed in several in vitro investigations [26,27,28]. To overcome the intrinsic ambiguity in using parameters measuring different physical quantities, some authors have suggested the insertion energy (IE) may be a more informative primary stability parameter [29,30]. IE, that is the total work needed to place the implant into its seat, may be more informative than the IT and the ISQ or the PTV. While these measure the stability of the implant after it has been placed, IE measures the implant stability while it is being placed, as a sum of all the interactions the implant has while descending into the pre-drilled tunnel. Indeed, in a study on bovine ribs, IE was shown to better detect the situations where sufficient primary stability can be achieved even in softer bone [29] when bone quality is classified according to Lekholm and Zarb [31]. IE might also detect primary stability enhancements provided by under preparation better than ISQ or IT measurements [32]. In a clinical setting, the IE may be measured as the integral of the torque-depth curve (I), a quantity differing from IE only by a multiplicative constant under the conditions that the implant threads are evenly spaced and the implant rotation speed is constant. These conditions are very often met in a clinical setting. (I) has been shown to be more sensitive to bone density variations than IT, ISQ, and the reverse torque (RT) both on polyurethane foam [33] and equine blocks [34]. (I) was also observed in human and bovine rib studies to correlate significantly with the bone to the implant contact (BIC) area [35,36]. On bovine ribs, (I) was found to discriminate between different implant geometries (cylindric, cylindric-modified, and tapered-cylindric) [37] and to detect different preparation conditions, namely undersizing and tapping, better [38]. On photoelastic resin blocks, mimicking D1 bone, (I) was shown to correlate significantly with the mechanical stress the material was subjected to when implants were placed [39]. When (I) was used in a study involving implants placed in low-density posterior maxillary sites concomitantly to sinus augmentation, it was observed it provided different information than IT concerning primary stability in relation to the residual ridge bone height (RBH) [40]. Overall, these studies suggested that to determine the implant primary stability achieved at insertion, measuring (I) at implant placement might be more useful, clinically, than measuring the IT and the ISQ. To date, no studies have determined whether (I) or IE detects variations in primary stability caused by changes in the implant length or diameter better than other primary stability measuring parameters. This study aimed to investigate this question on polyurethane foam blocks under conditions introducing the fewest confounding factors as possible.

## 2. Materials and Methods 

**Figure 1:**
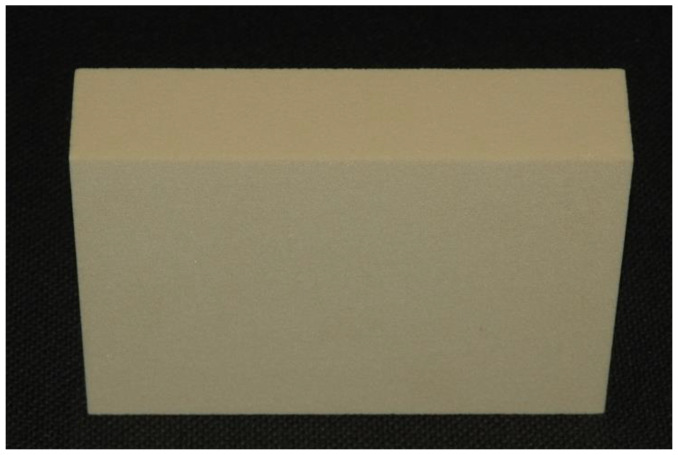
An example of the solid, monolithic rigid polyurethane 13 × 18 × 4 cm foam blocks used in the present study.

In this in vitro study, four homogeneous synthetic blocks were used, mimicking human bone. They were solid rigid polyurethane 13 × 18 × 4 cm foam blocks (Sawbones, Vashon Island, Washington, USA; Figure 1) produced as an alternative test medium for human cancellous/cortical bone. The blocks had different densities (0.26, 0.33, 0.49, and 0.65 g/cm^3^).

**Figure 2:**
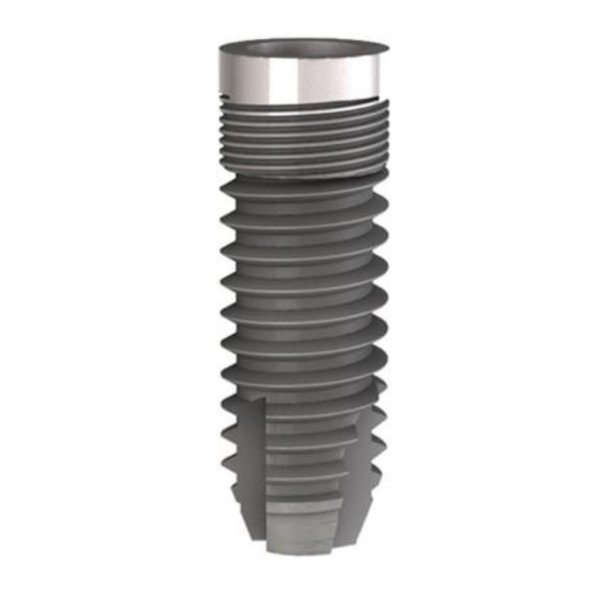
The implant used in the present study. The fixture is cylindrical and features a double-etched, sandblasted surface.

Even though polyurethane foam does not replicate the human bone structure, it does display mechanical properties in the range of human cancellous bone as described in the ASTM F-1839-08 standard [41]. The implants were cylindrical fixtures (Stone, IDI Evolution, Concorezzo, Italy) featuring a double-etched, sandblasted surface (Figure 2). These implants feature an evenly-threaded body and a micro-threaded head. Implants used in the present study had different diameters and lengths, as detailed in Table 1. Diameters and length were those available for this implant system. Ten implants for each size were placed into each block, according to the manufacturer’s instructions. Details about the final (body) and countersink drills diameters are also provided in Table 1. Implants were placed with no irrigation. 

An instantaneous torque-measuring micromotor (TMM2, IDI Evolution, Concorezzo, Italy) was used for primary stability measurement at the time of implant placement. The micromotor controlling console is sized 310 × 325 × 180 mm; the micromotor delivers 100 Ncm maximum torque. The medical touch screen of the controlling console displays the instantaneous torque with a 1-Ncm precision.

**Figure 3:**
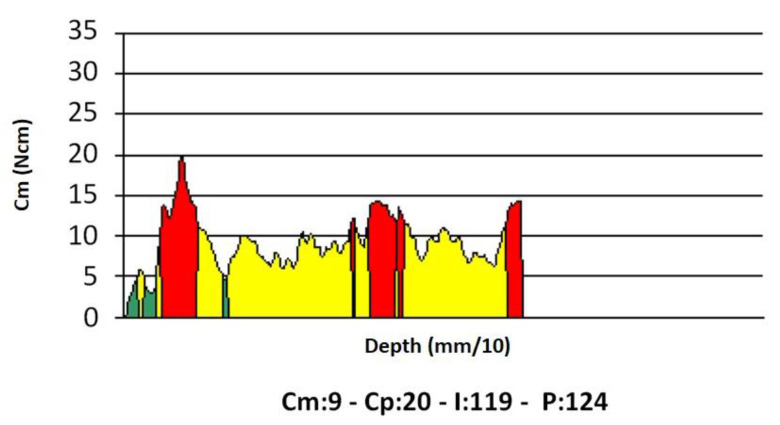
An example of a torque-depth plot generated from the micromotor measurements during implant insertion. Cm, average torque; Cp, peak torque (i.e., the insertion torque IT); (I), integral of the torque-depth curve; *p*, depth reached by the probe/implant in tenths of a millimeter.

Implant insertion was carried out at a preset rotation speed of 35 rpm. During measurement, the device displayed a torque/depth graph showing how the instantaneous torque varied according to the implant depth, together with the average torque (Cm), peak torque (Cp), and (I) (the torque-depth curve integral; Figure 3).

The Cp is commonly known as the IT. It should be noted that, under the conditions of the present study (the insertion speed constant, and evenly spaced implant threads), (I) was equal to IE multiplied by a constant factor [33]. A summary of the values provided by the micromotor is given in Table 2. All measurements were stored in the device’s solid-state memory and were later downloaded for statistical analysis.

### Data Analysis

IT and (I) data that were collected with each insertion condition were first checked for normality using the Shapiro–Wilk test. As their distribution was found to be normal, a single IT or (I) average value ± SD (standard deviation) was calculated for each insertion condition. These values were then used to draw IT-diameter, IT-length, (I)-diameter, and (I)-length plots. The correlation between the two variables, under each implant geometry and bone density condition, was studied by calculating the corresponding Pearson’s correlation coefficient and drawing the best fitting lines according to the least square method, assuming the relation between the two variables was linear (i.e., described by a y = mx + q equation, x and y being the two variables of interest). Site preparation according to the manufacturer instructions involves a certain degree of undersizing, that is preparing a final implant seat having a diameter smaller than that of the implant. Such undersizing is expected to have an effect both on IT and on I measurements. To investigate the effect of undersizing on the two stability parameters, IT and (I) data were also normalized by an undersizing coefficient C. This was calculated as C = (1 − r) = 1 − r_d_/r_i_ where r_d_ was the diameter of the final (body) drill, and r_i_ was the diameter of the implant threads (C was dependent on the specific insertion condition of interest; we omitted indexing it for the sake of clarity). Normalized IT and (I) data, from now on indicated as IT* and (I)*, were then calculated as IT* = IT/C and (I)* = (I)/C, and their error was calculated using the propagation of error formula [42]. All formulas used in the present study are summarized in Table 3.

The relation between IT*, (I)*, and the implant diameter or length was again investigated by calculating the corresponding Pearson’s correlation coefficients and assessing whether the IT*-diameter and (I)*-diameter, IT*-length, and (I)*-length plots could be best fitted by straight lines (i.e., if a linear relationship could be found between each pair of variables). The slopes of the best fitting lines were then plotted versus the block density to investigate how changes in bone (block) density affected the variation in primary stability consequent to a unitary (1 mm) change either in the fixture diameter or length.

All values in this work are the mean ± standard deviation (SD). Statistical calculations were performed using standard statistical software (Origin 2020, OriginLab, Northampton, MA, USA). The methodology was reviewed by an independent statistician.

## 3. Results

Data for each insertion condition are provided in Table 4. 

**Figure 4:**
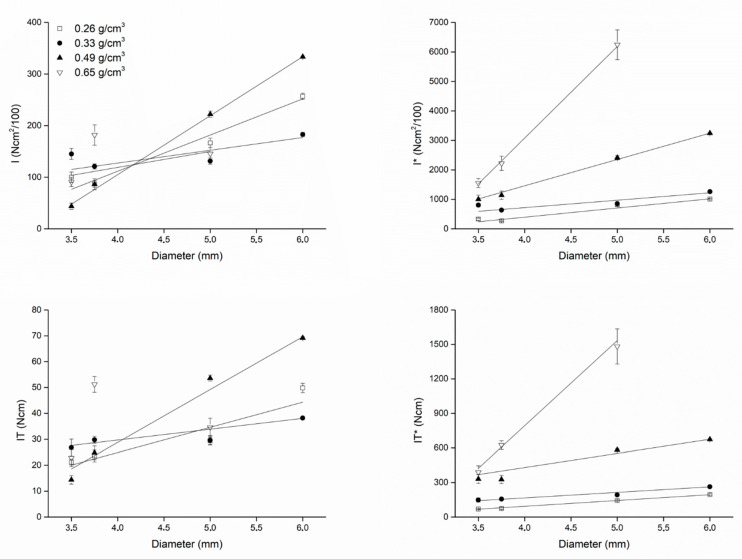
Plots describing how (I) and (I)* (top) and IT and IT* (bottom) varied as a function of the fixture diameter when implants were inserted in blocks with different densities. When (I) and IT were normalized for the undersizing coefficient (right), the effect of bone density on the correlation between (I)* or IT* and the fixture diameter could be appreciated: the greater the density, the greater (I)* or IT*. Such an effect could not be appreciated for the non-normalized variables (I) and IT, because of the effect of different site preparations (that is, different undersizing) when placing the implants according to the implant manufacturer’s instructions. The linear fit was better (i.e., the Pearson’s r coefficients were higher) for normalized variables than for non-normalized ones (see Table 5).

**Figure 5:**
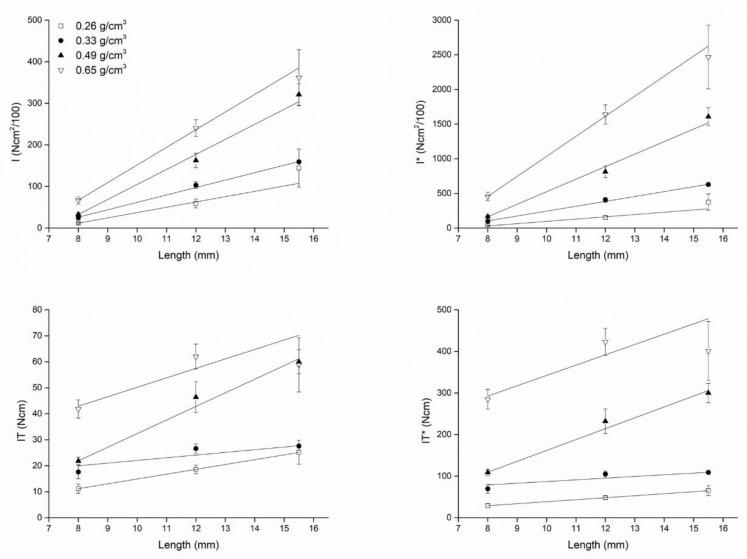
Plots show how (I) and (I)* (top) and IT and IT* (bottom) varied as a function of the fixture length when implants were inserted in blocks with different densities. The effect of bone density on the correlation between (I) or IT and the implant length could be appreciated even considering non-normalized variables (left): the greater the density, the greater (I) and IT. Such effect was equally observed when considering the normalized (I)* and IT* parameters (right). Regression lines fitted equally well (i.e., the Pearson’s r coefficients were similar) both the non-normalized and the normalized variables (see Table 5).

Plots showing how IT, (I), IT*, (I)* varied as a function of the fixture diameter and length are shown in Figure 4 (diameter) and Figure 5 (length).

The plots also show the best fitting lines to the experimental data, whose equations are provided in Table 5, together with the corresponding Pearson’s coefficients describing the correlation between IT, IT*, (I), and (I)* and the implant diameter and length. 

**Figure 6:**
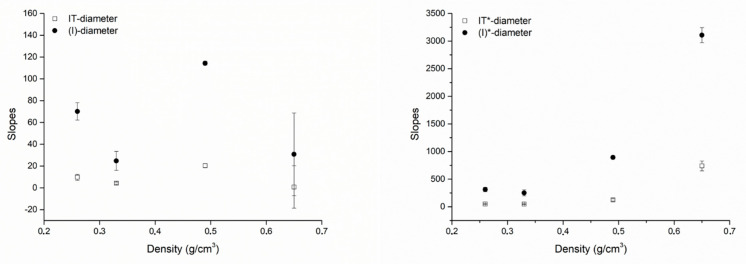
Plots showing how the slopes of the best fitting lines to the experimental data concerning the fixture diameters varied as a function of the block densities the fixtures were placed in. Left, slopes of the best fitting lines to the (I)-diameter and the IT-diameter plots; right, slopes of the lines best fitting the (I)*-diameter and the IT*-diameter plots. When (I) or IT were considered (left) they seemed to be not related to density in any way. When (I)* and IT* were considered (right), instead, a relation seemed to exist between bone density and them: the (I)*-diameter and IT*-diameter slopes appeared to vary as a non-linear function of bone density. Changes in (I)* plot slopes were always greater than those of the IT* plot slopes.

**Figure 7:**
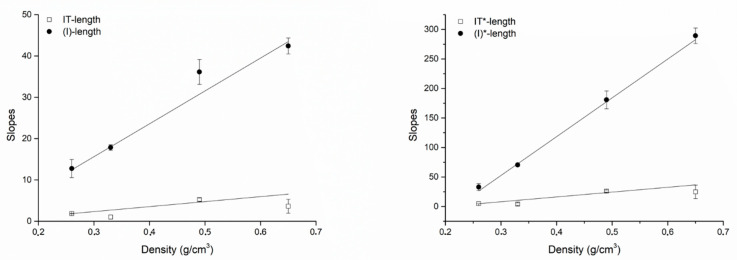
Plots showing how the slopes of the best fitting lines to the experimental data concerning the fixture length varied as a function of the block densities the fixtures were placed in. Left, slopes of the best fitting lines to the (I)-length and the IT-length plots; right, slopes of the best fitting lines to the (I)*-length and the IT*-length plots. Even with no correction for undersizing, the slopes always varied as a linear function of bone densities. Changes in (I) or (I)* plot slopes were always greater than those of IT or IT* plot slopes.

The correlation coefficients for IT* or (I)* and the fixture diameter were greater than the correlation coefficients for IT or (I) and the fixture diameter. However, the correlation coefficients for IT* or (I)* and the fixture length were not greater than the correlation coefficients for IT or (I) and the fixture length. The slopes of all best fitting lines to the experimental data were systematically greater considering (I) or (I)* compared to IT or IT*, indicating that a unitary change (1 mm) in the fixture diameter or length had a greater effect on (I) or (I)* than on IT or IT*. Figure 6 (diameter) and Figure 7 (length) show the angular coefficients (i.e., the slopes) of the best fitting lines to the experimental data plotted against density. Concerning the diameters, slopes describing how a unitary change (1 mm) in the fixture diameter affects primary stability seemed to not be a clear function of density. Concerning IT (r = 0.82944) and (I) (r = 0.81343), while they seemed to vary as a non-linear function of density both for IT* (r = 0.38289) and (I)* (r = 0.83095; Figure 6), a linear correlation was found, instead, between the IT (r = 088582), (I) (r = 0.99024), IT* (r = 0.94239) and (I)* (r = 0.99707) slopes describing how a unitary change (1 mm) in the fixture length affects primary stability and the block density (Figure 7). In all cases, the increase concerning (I)* was greater than that concerning IT*, indicating that a unitary change in density (1 g/cm^3^) causes a greater effect on (I)* than on IT*. When considering how the slopes concerning (I) or IT and length varied as a function of the block densities, this was also true for (I) when compared to IT.

## 4. Discussion

Results of the present study provide evidence that (I) at implant insertion might better detect changes in the implant diameter or length than IT. In all conditions that were assessed, in fact, the relation between (I) and the implant diameter or length was described by linear equations having a greater angular coefficient (slope) than those relating to IT. This means, in other words, that (I) is more sensitive than IT to changes in the implant diameter or length. It follows that, using (I) instead of IT to measure the implant primary stability in the clinical setting, the oral surgeon might gain a more precise and objective assessment of his/her choices concerning the size of the fixture being placed, which can actually allow the achievement of the desired primary implant stability. This should be the subject of further studies. 

The observation that (I) is more sensitive than IT to dimensional changes of the fixture, are consistent with the fact that (I) measures a cumulative quantity; i.e., the sum of all interactions the fixture had while descending into its tunnel, while IT measured only the maximum friction encountered during such descent [33]. These results, therefore, indicated that (I) might be more useful than IT to validate pre-surgical choices concerning implant dimensions aimed to achieve enhanced primary stability.

Results of the present study also suggested that, to appropriately assess the effect of a unitary increase in diameter on primary stability, the extent of undersizing, measured by the ratio between the final (body) drill diameter and the diameter of the fixture threads, needs to be taken into account. When IT or (I) measurements were corrected with the undersizing coefficient C, in fact, both the correlation coefficients between IT* or (I)* and the implant diameter were greater than those concerning IT or (I), indicating IT* and (I)* describe the relation between the implant diameter and its stability better. This finding was expected based on the observation that an increase in undersizing should be, on first approximation, physically equivalent to an increase in the fixture diameter. The C coefficient is calculated as the complement to 1 of the ratio between the implant thread diameter and the final (body) drill diameter. Given this ratio is constant, C will also be constant. Therefore, undersizing may be visualized, in the clinical setting, as having a simple linear effect on primary stability, determined by such a ratio. Conversely, the increase in primary stability that the oral surgeon should expect when increasing the implant diameter will be proportional to the diameter increase only if the coefficient C is constant. The effect of undersizing seems to be less relevant when considering how the fixture length affects its primary stability, as the correlation coefficients between IT, IT*, (I), (I)* and length observed in the present study were quite similar. This observation might be explained by considering the relative effect that a change in diameter or length has on the implant surface: as a first approximation, the implant may be regarded as a cylinder contacting bone through its lateral and bottom surfaces. Indicating the cylinder radius as R and its length L, the total area contacting bone would be A = πR^2^ + 2πRL = πR(R + 2L). Supposing R or L are increased by the same quantity *k*, an increase in diameter would provide an increase in area ΔA_diam_ = π(R + *k*)(R + *k* +2L) − πR(R + 2L) = π(*k*^2^ + 2 *k* R + 2 *k* L); an increase in length would instead provide a surface increase equal to ΔA_length_ = πR(R + 2(L+ *k*) − πR(R + 2L) = 2 π *k* R, with ΔA_diam_ − ΔA_length_ = π(*k*^2^ + 2 *k* R + 2 *k* L) − 2 π k R = π(*k*^2^ + 2 *k* L). It follows that an increase in the implant diameter will always provide a greater increase in the area contacting bone than an increase in its length, and that the surface increase will be proportional to the square of the increase in diameter. These considerations may also explain why a unitary increase in the fixture diameter stabilizes the fixture more, by rule, than an equal increase in length, as observed in the present study and by other authors in in vitro studies [43,44,45]. These observations suggested that using shorter and larger implants in a clinical setting may be as viable as, or more viable than, using longer ones; this piece of information may be useful when dealing with delicate anatomic structures, such as the inferior alveolar nerve or the maxillary sinus, and it is consistent with current clinical evidence concerning short implants [46,47,48]. These observations also provide theoretical support for the application of immediate loading protocols on short implants; indeed, the current clinical evidence indicates that the immediate loading of short implants is comparable to conventional length implants in terms of implant survival, marginal bone level change, and ISQ [49,50,51]. 

Results of the present study concerning the effect of bone density on changes in primary stability parameters caused by changes in the fixture diameter or length indicated that: (a) at higher densities, a given increase in the fixture diameter or length will provide a greater implant stabilization than at lower densities; (b) increases in primary stability caused by an increase in the fixture diameter and length are better detected using (I) or (I)* than when using IT or IT*; and (c) when densities are higher, the increase in primary stability (either measured by (I), (I)*, IT or IT*) that is achieved by increasing the implant length/diameter is more than proportional to the density increase, while it is proportional to the density increase when the fixture length is increased. This latter finding might be explained by considering greater densities as associated with greater friction levels around the implant. Such friction, when increasing the implant diameter, will increase proportionally to the increase in the area surface; that is, according to the square of the increase in diameter. Instead, the increase in friction due to an increase in density is expected to vary only linearly with an increase in the fixture length. These observations concerning the effect of bone density, when translated in the clinical setting, suggest that, when low bone density is encountered (as, for example, in the posterior maxilla), choosing larger rather than longer implants may be helpful to achieve greater implant stability. This indication is consistent with current clinical evidence [47,48,49,52].

The present study has several limitations. The first concerns tests of the biomaterials being used; while the blocks displayed some macroscopic features similar to human bone, there was no trabecular structure. The material’s elastic response to mechanical stress is therefore expected to be unlike that of human cancellous bone [34,53]. Indeed, Orlando et al. [34] showed that, while (I)-density plots are still linear when equine bone is used instead of polyurethane foam, the linear relation between these two quantities is described by a different equation. Further, data in the present study were collected under no irrigation; Di Stefano et al. [33] showed that, on polyurethane foam blocks, irrigation had virtually no effect on primary stability parameters, such as (I), IT, RT, and ISQ. However, the experiments described in the present study should be repeated under irrigation and on testing materials that better mimic human bone (such as, equine bone blocks, bovine ribs or cadaver bone). Moreover, the present study compared (I) and (I)* to IT and IT*, respectively, but not to ISQ. The comparison between (I) and ISQ, as far as changes in the fixture length and diameter are concerned, should be the subject of additional investigations.

Finally, it is known that (I) and IT vary according to bone density depending on the implant shape and other macro- and micro-morphological characteristics [37]. Accordingly, primary stability values as well as equations and correlation coefficients calculated in the present study should be regarded as valid only for the specific implant model that was used to perform the experiments. While the overall picture that emerges from the present study; i.e., that (I) and (I)* are more sensitive to changes in the fixture diameter and length than IT and IT*, and that increasing the implant diameter stabilizes the implant more than increasing its length, different implant models and brands are expected to be characterized by different (I)-, (I)*-, IT-, and IT-diameter/length plots.

## 5. Conclusions

(I), as a primary stability measuring parameter, is more sensitive than IT to changes in stability that can be achieved by increasing the fixture diameter or length. When used to assess changes in stability given by varying the fixture diameter, (I) should be normalized for undersizing, as undersizing increases primary stability proportionally to (1-r), with r being the ratio between the final (body) drill diameter and that of the implant threads. All other conditions being the same, an increase in the implant diameter stabilizes the fixture more than an equal increase in length.

## Figures and Tables

**Table 1:** Sizes of implants used in the present study. To assess how changes in the fixture diameter affected either IT or (I), 12-mm long implants were used, featuring four different (thread) diameters: 3.5, 3.75, 5.0 and 6.0 mm. To assess the effect of length changes, 3.75-mm (thread) wide implants were used with lengths of 8.0, 12.0 or 15.5 mm.

Block Density (g/cm^3^)	Implant Size (Thread Diameter × Length, mm)	Final Drill Diameter (mm)	Countersink Drill Diameter (mm)
0.26	3.5 × 12.0	2.8	None
	3.75 × 8.0	3.0	3.5
	3.75 × 12.0	3.0	3.5
	3.75 × 15.5	3.0	3.5
	5.0 × 12.0	3.8	4.3
	6.0 × 12.0	4.2	5.3
0.33	3.5 × 12.0	2.8	None
	3.75 × 8.0	2.8	3.7
	3.75 × 12.0	2.8	3.7
	3.75 × 15.5	2.8	3.7
	5.0 × 12.0	4.0	4.8
	6.0 × 12.0	4.8	6.0
0.49	3.5 × 12.0	3.2	3.5
	3.75 × 8.0	3.2	4.0
	3.75 × 12.0	3.2	4.0
	3.75 × 15.5	3.2	4.0
	5.0 × 12.0	4.2	5.0
	6.0 × 12.0	5.0	6.0
0.65	3.5 × 12.0	3.2	3.5
	3.75 × 8.0	3.2	4.0
	3.75 × 12.0	3.2	4.0
	3.75 × 15.5	3.2	4.0
	5.0 × 12.0	4.8	5.0
	6.0 × 12.0	5.0	6.0

**Table 2:** A summary of the measures provided by the micromotor.

Measure	Symbol as Provided by the Micromotor	Unit of Measure	Information Provided
Average torque	Cm	Ncm	When recorded at probing, it is a quantification of bone density.
Peak torque	Cp	Ncm	When recorded at implant insertion, it is the maximum torque that was exerted by the micromotor during implant placement. In the present work, it has been indicated by the acronym IT (insertion torque).
Integral	I	Ncm^2^/100	When measured at implant insertion, it provides the area bounded by the torque-depth curve (Figure 3). If the implant threads are evenly spaced and the rotation speed at insertion is constant (two conditions that are met in this study), the integral (I) is equal to the insertion energy, IE, multiplied by a constant factor.
Depth	*p*	mm/10	Indicates the depth reached by the probe, when density is being measured, or by the implant when it is being placed.

**Table 3:** Formulas used in the present study.

Quantity	Formula
Undersizing coefficient, C	C = (1 − r) = 1 − r_d_/r_i_ where r_d_: final (body) drill diameter r_i_: implant threads diameter
Normalized IT, IT*	IT* = IT/C
Normalized (I), (I)*	(I)* = I/C
Propagation of error formula [42]	σ^2^_y_ = (df/dx_1_)^2^σ^2^_1_ + … + (df/dx_n_)^2^σ^2^_n_ where y = f(x_1_, … x_n_) is a function of multiple variables and σ_y_ is the standard deviation of y σ_i_ for (i = 1 … n) is the standard deviation of the x_i_ independent variable
Standard deviation of IT*, σ_IT_*	σ_IT_* = σ_IT_/C (being σ_c_ = 0)
Standard deviation of (I)*, σ_(I)_*	σ_(I)_* = σ_(I)_/C (being σ_c_ = 0)

**Table 4:** Implant primary stability parameters corresponding to each insertion condition.

Diameter (mm)	Bone Density (g/cm^3^)							
	0.26		0.33		0.49		0.65	
	IT	IT*	IT	IT*	IT	IT*	IT	IT*
3.5	21.20 ± 1.10	26.80 ± 3.27	14.40 ± 1.67	22.80 ± 3.27	70.00 ± 3.62	148.89 ± 18.17	330.49 ± 38.40	389.27 ± 55.85
3.75	23.60 ± 2.30	29.80 ± 1.30	24.80 ± 2.68	51.20 ± 3.03	73.98 ± 7.22	156.84 ± 6.86	327.27 ± 35.41	625.24 ± 37.04
5.0	29.40 ± 1.34	29.60 ± 1.82	53.60 ± 1.14	34.60 ± 3.57	144.12 ± 6.58	193.04 ± 11.85	582.61 ± 12.39	1482.86 ± 152.80
6.0	49.80 ± 1.79	38.20 ± 0.45	69.20 ± 0.45	--	195.29 ± 7.02	263.45 ± 3.08	673.30 ± 4.35	--
	**(I)**	(I)*	**(I)**	(I)*	**(I)**	(I)*	**(I)**	(I)*
3.5	100.20 ± 9.98	145.20 ± 10.83	43.80 ± 6.10	91.00 ± 9.17	330.85 ± 32.97	806.67 ± 60.14	1005.25 ± 139.98	1553.66 ± 156.48
3.75	87.00 ± 6.28	121.00 ± 4.95	86.40 ± 10.53	182.00 ± 19.74	272.73 ± 19.70	636.84 ± 26.05	1140.18 ± 138.91	2222.52 ± 241.01
5.0	166.60 ± 9.13	131.40 ± 5.86	222.00 ± 6.32	145.70 ± 11.81	816.67 ± 44.74	856.96 ± 38.20	2413.04 ± 68.75	6244.29 ± 506.31
6.0	256.80 ± 6.14	182.80 ± 3.96	333.60 ± 1.14	--	1007.06 ± 24.08	1260.69 ± 27.33	3245.84 ± 11.09	--
**Length (mm)**	**IT**	IT*	**IT**	IT*	**IT**	IT*	**IT**	IT*
8.0	11.20 ± 1.79	17.60 ± 2.61	21.80 ± 1.48	41.80 ± 3.49	28.97 ± 4.63	69.47 ± 10.29	109.00 ± 7.42	285.00 ± 23.81
12.0	18.60 ± 1.67	26.60 ± 1.82	46.40 ± 5.94	62.00 ± 4.80	48.10 ± 4.33	105.00 ± 7.17	232.00 ± 29.71	422.73 ± 32.70
15.5	25.20 ± 4.60	27.60 ± 0.55	60.00 ± 4.64	58.80 ± 10.43	65.17 ± 11.91	108.95 ± 2.16	300.00 ± 23.18	400.91 ± 71.09
	**(I)**	(I)*	**(I)**	(I)*	**(I)**	(I)*	**(I)**	(I)*
8.0	11.80 ± 1.79	25.00 ± 5.61	33.00 ± 3.74	66.60 ± 8.88	30.52 ± 4.63	98.68 ± 22.15	165.00 ± 18.71	454.09 ± 60.52
12.0	59.00 ± 10.75	103.00 ± 8.37	162.60 ± 17.18	240.60 ± 20.18	152.59 ± 27.79	406.58 ± 33.03	813.00 ± 85.92	1640.45 ± 137.60
15.5	144.40 ± 45.87	159.20 ± 4.44	321.20 ± 25.66	361.80 ± 67.34	373.45 ± 118.64	628.42 ± 17.52	1606.00 ± 128.28	2466.82 ± 459.14

**Table 5:** Coefficients (m, slope; q, intercept) of the y = mx + q best fitting lines to the implant primary stability data (IT, IT*, (I), (I)*) when plotted versus either the fixture diameter or length, for each block density. The table also shows the corresponding Pearson’s r linear correlation coefficients.

Plot	Density (g/cm^3^)	m (Slope)	q (Intercept)	r (Pearson’s)
IT-Diameter	0.26	9.71 ± 2.71	−13.94 ± 12.16	0.93003
	0.33	4.17 ± 0.99	13.03 ± 5.70	0.94774
	0.49	20.41 ± 1.90	−52.86 ± 10.86	0.99148
	0.65	0.81 ± 19.45	33.83 ± 79.06	0.04176
IT*-Diameter	0.26	50.49 ± 2.18	−108.29 ± 9.28	0.99814
	0.33	48.00 ± 4.15	−25.38 ± 23.28	0.99261
	0.49	122.65 ± 20.63	−60.48 ± 120.77	0.97285
	0.65	740.11 ± 89.34	−2167.77 ± 333.79	0.99279
(I)-Diameter	0.26	70.14 ± 7.86	−168.86 ± 38.08	0.98767
	0.33	24.75 ± 8.77	28.52 ± 44.72	0.89414
	0.49	114.34 ± 1.84	−352.37 ± 10.85	0.99974
	0.65	30.82 ± 37.99	−4.17 ± 155.17	0.63003
(I*)-Diameter	0.26	311.62 ± 38.67	−850.46 ± 178.62	0.98495
	0.33	251.49 ± 54.47	−285.72 ± 265.16	0.95615
	0.49	892.91 ± 28.47	−2110.76 ± 169.51	0.99898
	0.65	3106.65 ± 137.61	−9347.63 ± 506.67	0.99902
IT-Length	0.26	1.86 ± 0.01	−3.67 ± 0.12	0.99998
	0.33	1.03 ± 0.51	11.75 ± 7.62	0.89729
	0.49	5.23 ± 0.40	−19.97 ± 3.66	0.99709
	0.65	3.64 ± 1.68	13.81 ± 16.97	0.90775
IT*-Length	0.26	4.80 ± 0.03	−9.49 ± 0.30	0.99998
	0.33	4.07 ± 2.00	46.37 ± 30.07	0.89729
	0.49	26.17 ± 2.00	−99.83 ± 18.30	0.99709
	0.65	24.81 ± 11.46	94.18 ± 115.71	0.90775
(I)-Length	0.26	12.76 ± 2.19	−90.35 ± 17.88	0.98554
	0.33	17.85 ± 0.67	−116.65 ± 8.74	0.99929
	0.49	36.15 ± 3.02	−256.49 ± 25.43	0.99654
	0.65	42.43 ± 1.93	−272.47 ± 17.15	0.99897
(I*)-Length	0.26	33.01 ± 5.68	−233.66 ± 46.25	0.98554
	0.33	70.46 ± 2.66	−460.47 ± 34.50	0.99929
	0.49	180.73 ± 15.08	−1282.47 ± 127.15	0.99654
	0.65	289.28 ± 13.13	−1857.72 ± 116.95	0.99897
